# 5α-Pregna-1,20-dien-3-one

**DOI:** 10.1107/S1600536810000863

**Published:** 2010-01-13

**Authors:** Guang-ying Chen, Mei-Yan Wei, Ni Tan, Zhen Liu, Rui-Yun Yang

**Affiliations:** a Hainan Provincial Key Laboratory of Tropical Pharmaceutical Herb Chemistry, College of Chemistry and Chemical Engineering, Hainan Normal University, Haikou, Hainan 571158, People’s Republic of China; b School of Pharmacy, Guangdong Medical College, Dongguan, Guangdong 523808, People’s Republic of China; c School of Chemistry and Chemical Engineering, University of South China, Hengyang, Hunan 421001, People’s Republic of China; d College of Chemistry and Chemical Engineering, Luoyang Normal University, Luoyang, Henan 471022, People’s Republic of China; e Key Laboratory for the Chemistry and Molecular Engineering of Medicinal Resources, Ministry of Education, School of Chemistry and Chemical Engineering, Guangxi Normal University, Guilin, Guangxi 541004, People’s Republic of China

## Abstract

The title compound, C_21_H_30_O, was isolated from the soft coral *Sinularia* sp. The mol­ecule contains four alicyclic rings, all *trans*-fused, among which three six-membered rings are in different distorted chair conformations while a five-membered ring assumes an envelope form.

## Related literature

For general background to marine pregnanes isolated from marine organisms, see: Higgs & Faulkner (1977 ▶); Blackman *et al.* (1985 ▶); Hooper & Davies-Coleman (1995 ▶); Kittakoop *et al.* (1999 ▶); Li *et al.* (2009 ▶); Yan *et al.* (2004 ▶, 2007 ▶); Zhang *et al.* (2005 ▶); Seo *et al.* (1995 ▶).
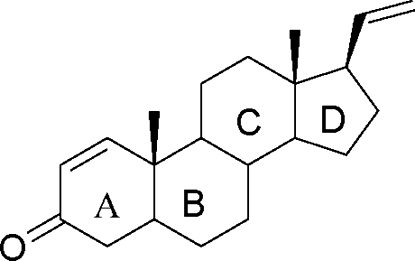


## Experimental

### 

#### Crystal data

C_21_H_30_O
*M* *_r_* = 298.45
Orthorhombic, 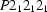

*a* = 7.2619 (13) Å
*b* = 10.998 (2) Å
*c* = 21.964 (4) Å
*V* = 1754.2 (6) Å^3^
*Z* = 4
Mo *K*α radiation
μ = 0.07 mm^−1^
*T* = 293 K
0.25 × 0.22 × 0.20 mm

#### Data collection

Bruker APEXII CCD diffractometer
Absorption correction: multi-scan (*SADABS*; Sheldrick, 1996 ▶) *T*_min_ = 0.984, *T*_max_ = 0.987
7653 measured reflections
1995 independent reflections
1563 reflections with *I* > 2σ(*I*)
*R*_int_ = 0.053

#### Refinement

*R*[*F* ^2^ > 2σ(*F* ^2^)] = 0.051
*wR*(*F* ^2^) = 0.114
*S* = 1.10
1995 reflections
201 parameters
H-atom parameters constrained
Δρ_max_ = 0.13 e Å^−3^
Δρ_min_ = −0.18 e Å^−3^

### 

Data collection: *APEX2* (Bruker, 2004 ▶); cell refinement: *SAINT* (Bruker, 2004 ▶); data reduction: *SAINT*; program(s) used to solve structure: *SHELXS97* (Sheldrick, 2008 ▶); program(s) used to refine structure: *SHELXL97* (Sheldrick, 2008 ▶); molecular graphics: *SHELXTL* (Sheldrick, 2008 ▶) and *DIAMOND* (Brandenburg, 2006 ▶); software used to prepare material for publication: *SHELXTL*.

## Supplementary Material

Crystal structure: contains datablocks global, I. DOI: 10.1107/S1600536810000863/gk2251sup1.cif

Structure factors: contains datablocks I. DOI: 10.1107/S1600536810000863/gk2251Isup2.hkl

Additional supplementary materials:  crystallographic information; 3D view; checkCIF report

## Figures and Tables

**Fig. 1.**
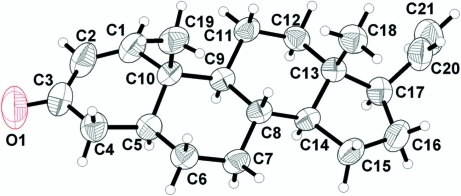
View of the title molecular structure with atom numbering scheme and 30% probability displacement ellipsoids for non-hydrogen atoms.

## supplementary crystallographic information

### Comment

Soft corals have been well recognized as marine organisms containing large quantities of secondary metabolites that exhibit various biological activities. In this regard, 5α-pregna-1,20-dien-3-one was firstly isolated as a marine natural product from an unknown coral (Higgs *et al.*, 1977), subsequently from a soft coral of the genus *Capnella* (Blackman *et al.*, 1985; Hooper *et al.*, 1995), *Scleronephthya pallida* (Kittakoop *et al.*, 1999), *Scleronephthya sp.* (Yan *et al.*, 2004), *Spongodes sp.* (Yan *et al.*, 2004; Yan *et al.*, 2007), all of those belong to the family Nephtheidae. Furthermore, the title compound was also reported to be isolated from the family Alcyoniidae from two soft corals *Sinularia papillosa* (Zhang *et al.*, 2005) and *Alcyonium gracillimun*(Seo *et al.*, 1995). In course of our investigations of bioactive substances from marine organisms (Li *et al.*, 2009), a soft coral *Sinularia sp*. which was collected from Sanya, was studied. In this paper, we describe the isolation, structure elucidation and crystal structure of the title compound.

The molecular structure of the title compound is shown in Fig. 1. The four fused rings are in different distorted conformations. Due to the C1=C2 double bond, ring A is highly distorted with a half-chair conformation. Rings B and C have slightly flattened chair conformations. Ring D assumes an unusual envelope conformation, probably induced by the vinyl substituent. Sstablization of the crystal structure is due only to weak van der Waals interactions.

### Experimental

The soft coral *Sinularia sp*. was collected by SCUBA diving off coral reef at a depth of 15–20 m at Sanya in Hainan Island, PR China, in June 2005. The sample was frozen immediately after collection. The species was identified by Professor Renlin Zou (South China Sea Institute of Oceanology, Chinese Academy of Sciences). The soft coral (800 g, wet weight) was homogenized and extracted with MeOH for three times at room temperature, and the MeOH extracts were combined and then concentrated under vacuo to give a dark brown residue (25.6 g). The residue was partitioned between H_2_O and EtOAc. The ethyl acetate fraction was subjected to column chromatography over silica gel and Sephadex LH-20 to give the pure title compound (54.9 mg). The crystalline compound was obtained through the slow evaporation of the ethyl acetate solution of the title compound.

### Refinement

All H atoms were positioned geometrically and treated as riding, with C—H bond lengths constrained to 0.93 Å (CH), 0.97 Å (CH_2_) 0.96 Å (methyl CH_3_), and with U_iso_(H) = 1.2U_eq_(C) or 1.5U_eq_(methyl).

### Crystal data

**Table:**

C_21_H_30_O	*F*(000) = 656
*M**_r_* = 298.45	*D*_x_ = 1.130 Mg m^−^^3^
Orthorhombic, *P*2_1_2_1_2_1_	Mo *K*α radiation, λ = 0.71073 Å
Hall symbol: P 2ac 2ab	Cell parameters from 7653 reflections
*a* = 7.2619 (13) Å	θ = 2.1–26.0°
*b* = 10.998 (2) Å	µ = 0.07 mm^−^^1^
*c* = 21.964 (4) Å	*T* = 293 K
*V* = 1754.2 (6) Å^3^	Block, colorless
*Z* = 4	0.25 × 0.22 × 0.20 mm

### Data collection

**Table:**

Bruker APEXII CCD diffractometer	1995 independent reflections
Radiation source: fine-focus sealed tube	1563 reflections with *I* > 2σ(*I*)
graphite	*R*_int_ = 0.053
Detector resolution: 0 pixels mm^-1^	θ_max_ = 26.0°, θ_min_ = 2.1°
φ and ω scans	*h* = −8→6
Absorption correction: multi-scan (*SADABS*; Sheldrick, 1996)	*k* = −13→13
*T*_min_ = 0.984, *T*_max_ = 0.987	*l* = −27→25
7653 measured reflections	

### Refinement

**Table:**

Refinement on *F*^2^	Primary atom site location: structure-invariant direct methods
Least-squares matrix: full	Secondary atom site location: difference Fourier map
*R*[*F*^2^ > 2σ(*F*^2^)] = 0.051	Hydrogen site location: inferred from neighbouring sites
*wR*(*F*^2^) = 0.114	H-atom parameters constrained
*S* = 1.10	*w* = 1/[σ^2^(*F*_o_^2^) + (0.0531*P*)^2^] where *P* = (*F*_o_^2^ + 2*F*_c_^2^)/3
1995 reflections	(Δ/σ)_max_ < 0.001
201 parameters	Δρ_max_ = 0.13 e Å^−^^3^
0 restraints	Δρ_min_ = −0.17 e Å^−^^3^

### Special details

**Table:**

Geometry. All e.s.d.'s (except the e.s.d. in the dihedral angle between two l.s. planes) are estimated using the full covariance matrix. The cell e.s.d.'s are taken into account individually in the estimation of e.s.d.'s in distances, angles and torsion angles; correlations between e.s.d.'s in cell parameters are only used when they are defined by crystal symmetry. An approximate (isotropic) treatment of cell e.s.d.'s is used for estimating e.s.d.'s involving l.s. planes.
Refinement. Refinement of *F*^2^ against ALL reflections. The weighted *R*-factor *wR* and goodness of fit *S* are based on *F*^2^, conventional *R*-factors *R* are based on *F*, with *F* set to zero for negative *F*^2^. The threshold expression of *F*^2^ > σ(*F*^2^) is used only for calculating *R*-factors(gt) *etc*. and is not relevant to the choice of reflections for refinement. *R*-factors based on *F*^2^ are statistically about twice as large as those based on *F*, and *R*- factors based on ALL data will be even larger.

### Fractional atomic coordinates and isotropic or equivalent isotropic displacement parameters (Å^2^)

**Table:**

	*x*	*y*	*z*	*U*_iso_*/*U*_eq_	
C1	0.4468 (4)	0.1678 (3)	0.18505 (11)	0.0607 (8)	
H1	0.5613	0.1344	0.1760	0.073*	
C2	0.4398 (5)	0.2554 (3)	0.22674 (13)	0.0709 (10)	
H2	0.5495	0.2815	0.2442	0.085*	
C3	0.2683 (5)	0.3124 (3)	0.24637 (12)	0.0684 (9)	
C4	0.0964 (4)	0.2700 (3)	0.21577 (12)	0.0602 (8)	
H4A	0.0108	0.3375	0.2127	0.072*	
H4B	0.0391	0.2075	0.2405	0.072*	
C5	0.1328 (4)	0.2193 (2)	0.15234 (11)	0.0470 (7)	
H5	0.1822	0.2870	0.1284	0.056*	
C6	−0.0413 (4)	0.1780 (3)	0.11961 (11)	0.0507 (7)	
H6A	−0.1328	0.2422	0.1210	0.061*	
H6B	−0.0918	0.1071	0.1399	0.061*	
C7	0.0026 (4)	0.1469 (2)	0.05360 (11)	0.0500 (7)	
H7A	−0.1075	0.1149	0.0344	0.060*	
H7B	0.0363	0.2210	0.0324	0.060*	
C8	0.1565 (3)	0.0555 (2)	0.04637 (11)	0.0417 (6)	
H8	0.1119	−0.0234	0.0610	0.050*	
C9	0.3289 (3)	0.0902 (2)	0.08406 (11)	0.0414 (6)	
H9	0.3736	0.1667	0.0666	0.050*	
C10	0.2826 (4)	0.1193 (2)	0.15166 (11)	0.0446 (6)	
C11	0.4847 (4)	−0.0019 (2)	0.07458 (11)	0.0500 (7)	
H11A	0.4480	−0.0796	0.0916	0.060*	
H11B	0.5932	0.0255	0.0964	0.060*	
C12	0.5332 (4)	−0.0188 (2)	0.00707 (11)	0.0477 (7)	
H12A	0.5836	0.0564	−0.0089	0.057*	
H12B	0.6266	−0.0814	0.0032	0.057*	
C13	0.3649 (3)	−0.0546 (2)	−0.02996 (11)	0.0430 (6)	
C14	0.2141 (3)	0.0410 (2)	−0.01977 (11)	0.0429 (6)	
H14	0.2689	0.1190	−0.0315	0.051*	
C15	0.0723 (4)	0.0120 (3)	−0.06889 (11)	0.0591 (8)	
H15A	0.0036	0.0842	−0.0802	0.071*	
H15B	−0.0133	−0.0498	−0.0551	0.071*	
C16	0.1885 (4)	−0.0347 (3)	−0.12272 (13)	0.0706 (9)	
H16A	0.1441	−0.1135	−0.1360	0.085*	
H16B	0.1811	0.0215	−0.1567	0.085*	
C17	0.3882 (4)	−0.0446 (3)	−0.09989 (11)	0.0528 (7)	
H17	0.4484	0.0334	−0.1083	0.063*	
C18	0.3017 (4)	−0.1838 (2)	−0.01350 (13)	0.0591 (8)	
H18A	0.4009	−0.2398	−0.0204	0.089*	
H18B	0.1985	−0.2059	−0.0384	0.089*	
H18C	0.2664	−0.1863	0.0286	0.089*	
C19	0.2221 (5)	0.0030 (3)	0.18588 (12)	0.0617 (9)	
H19A	0.3250	−0.0512	0.1895	0.093*	
H19B	0.1252	−0.0363	0.1636	0.093*	
H19C	0.1785	0.0245	0.2257	0.093*	
C20	0.5016 (5)	−0.1414 (3)	−0.12920 (12)	0.0672 (9)	
H20	0.4512	−0.2189	−0.1317	0.081*	
C21	0.6668 (5)	−0.1253 (4)	−0.15167 (14)	0.0843 (12)	
H21A	0.7218	−0.0490	−0.1500	0.101*	
H21B	0.7292	−0.1903	−0.1693	0.101*	
O1	0.2658 (4)	0.3879 (3)	0.28688 (11)	0.1084 (10)	

### Atomic displacement parameters (Å^2^)

**Table:**

	*U*^11^	*U*^22^	*U*^33^	*U*^12^	*U*^13^	*U*^23^
C1	0.052 (2)	0.083 (2)	0.0466 (16)	−0.0024 (18)	−0.0062 (13)	−0.0025 (15)
C2	0.061 (2)	0.096 (3)	0.0553 (18)	−0.013 (2)	−0.0124 (15)	−0.0138 (18)
C3	0.079 (2)	0.080 (2)	0.0458 (16)	−0.006 (2)	−0.0011 (16)	−0.0092 (16)
C4	0.064 (2)	0.069 (2)	0.0477 (17)	0.0012 (17)	0.0080 (15)	−0.0002 (14)
C5	0.0485 (17)	0.0497 (16)	0.0428 (14)	−0.0039 (14)	0.0042 (12)	0.0054 (11)
C6	0.0385 (16)	0.0609 (17)	0.0528 (15)	0.0040 (15)	0.0040 (12)	0.0011 (13)
C7	0.0376 (15)	0.0606 (17)	0.0519 (15)	0.0020 (15)	−0.0075 (12)	0.0021 (12)
C8	0.0352 (15)	0.0439 (15)	0.0461 (14)	−0.0050 (13)	−0.0032 (11)	0.0046 (11)
C9	0.0386 (15)	0.0432 (15)	0.0425 (13)	−0.0045 (13)	−0.0013 (11)	0.0055 (11)
C10	0.0430 (16)	0.0523 (15)	0.0386 (13)	−0.0017 (14)	−0.0032 (12)	0.0046 (11)
C11	0.0402 (16)	0.0606 (18)	0.0493 (15)	0.0031 (14)	−0.0084 (12)	−0.0012 (13)
C12	0.0408 (17)	0.0443 (15)	0.0580 (16)	0.0010 (13)	−0.0012 (12)	−0.0028 (12)
C13	0.0400 (16)	0.0423 (15)	0.0468 (15)	−0.0025 (13)	−0.0002 (12)	0.0019 (11)
C14	0.0392 (15)	0.0463 (15)	0.0432 (14)	−0.0017 (13)	−0.0053 (12)	0.0050 (11)
C15	0.0547 (18)	0.074 (2)	0.0490 (17)	0.0044 (17)	−0.0140 (14)	−0.0022 (14)
C16	0.072 (2)	0.092 (2)	0.0473 (16)	0.003 (2)	−0.0123 (16)	−0.0092 (16)
C17	0.0557 (19)	0.0532 (17)	0.0494 (16)	−0.0035 (16)	0.0026 (13)	−0.0025 (13)
C18	0.061 (2)	0.0501 (17)	0.0664 (17)	−0.0050 (16)	−0.0004 (15)	0.0007 (14)
C19	0.071 (2)	0.0659 (19)	0.0482 (16)	0.0009 (18)	0.0012 (14)	0.0170 (13)
C20	0.075 (2)	0.077 (2)	0.0502 (17)	0.000 (2)	0.0013 (16)	−0.0143 (15)
C21	0.071 (3)	0.108 (3)	0.074 (2)	0.013 (2)	0.009 (2)	−0.027 (2)
O1	0.114 (2)	0.125 (2)	0.0864 (17)	0.003 (2)	−0.0065 (16)	−0.0523 (16)

### Geometric parameters (Å, °)

**Table:**

C1—C2	1.330 (4)	C11—H11B	0.9700
C1—C10	1.498 (4)	C12—C13	1.520 (3)
C1—H1	0.9300	C12—H12A	0.9700
C2—C3	1.460 (5)	C12—H12B	0.9700
C2—H2	0.9300	C13—C18	1.536 (4)
C3—O1	1.217 (3)	C13—C14	1.534 (4)
C3—C4	1.493 (4)	C13—C17	1.549 (3)
C4—C5	1.524 (4)	C14—C15	1.525 (3)
C4—H4A	0.9700	C14—H14	0.9800
C4—H4B	0.9700	C15—C16	1.541 (4)
C5—C6	1.523 (4)	C15—H15A	0.9700
C5—C10	1.547 (4)	C15—H15B	0.9700
C5—H5	0.9800	C16—C17	1.539 (4)
C6—C7	1.523 (4)	C16—H16A	0.9700
C6—H6A	0.9700	C16—H16B	0.9700
C6—H6B	0.9700	C17—C20	1.492 (4)
C7—C8	1.512 (3)	C17—H17	0.9800
C7—H7A	0.9700	C18—H18A	0.9600
C7—H7B	0.9700	C18—H18B	0.9600
C8—C14	1.520 (3)	C18—H18C	0.9600
C8—C9	1.549 (3)	C19—H19A	0.9600
C8—H8	0.9800	C19—H19B	0.9600
C9—C11	1.532 (3)	C19—H19C	0.9600
C9—C10	1.556 (3)	C20—C21	1.309 (4)
C9—H9	0.9800	C20—H20	0.9300
C10—C19	1.547 (4)	C21—H21A	0.9300
C11—C12	1.535 (4)	C21—H21B	0.9300
C11—H11A	0.9700		
			
C2—C1—C10	124.4 (3)	H11A—C11—H11B	107.9
C2—C1—H1	117.8	C13—C12—C11	111.3 (2)
C10—C1—H1	117.8	C13—C12—H12A	109.4
C1—C2—C3	123.2 (3)	C11—C12—H12A	109.4
C1—C2—H2	118.4	C13—C12—H12B	109.4
C3—C2—H2	118.4	C11—C12—H12B	109.4
O1—C3—C2	121.5 (3)	H12A—C12—H12B	108.0
O1—C3—C4	122.0 (3)	C12—C13—C18	110.7 (2)
C2—C3—C4	116.5 (2)	C12—C13—C14	108.6 (2)
C3—C4—C5	112.4 (2)	C18—C13—C14	112.7 (2)
C3—C4—H4A	109.1	C12—C13—C17	115.1 (2)
C5—C4—H4A	109.1	C18—C13—C17	109.4 (2)
C3—C4—H4B	109.1	C14—C13—C17	100.0 (2)
C5—C4—H4B	109.1	C8—C14—C15	120.8 (2)
H4A—C4—H4B	107.9	C8—C14—C13	114.1 (2)
C6—C5—C4	113.4 (2)	C15—C14—C13	103.6 (2)
C6—C5—C10	111.5 (2)	C8—C14—H14	105.7
C4—C5—C10	113.0 (2)	C15—C14—H14	105.7
C6—C5—H5	106.1	C13—C14—H14	105.7
C4—C5—H5	106.1	C14—C15—C16	104.1 (2)
C10—C5—H5	106.1	C14—C15—H15A	110.9
C5—C6—C7	110.0 (2)	C16—C15—H15A	110.9
C5—C6—H6A	109.7	C14—C15—H15B	110.9
C7—C6—H6A	109.7	C16—C15—H15B	110.9
C5—C6—H6B	109.7	H15A—C15—H15B	109.0
C7—C6—H6B	109.7	C17—C16—C15	106.8 (2)
H6A—C6—H6B	108.2	C17—C16—H16A	110.4
C8—C7—C6	113.8 (2)	C15—C16—H16A	110.4
C8—C7—H7A	108.8	C17—C16—H16B	110.4
C6—C7—H7A	108.8	C15—C16—H16B	110.4
C8—C7—H7B	108.8	H16A—C16—H16B	108.6
C6—C7—H7B	108.8	C20—C17—C16	115.5 (3)
H7A—C7—H7B	107.7	C20—C17—C13	116.0 (2)
C7—C8—C14	111.9 (2)	C16—C17—C13	103.0 (2)
C7—C8—C9	112.2 (2)	C20—C17—H17	107.3
C14—C8—C9	108.3 (2)	C16—C17—H17	107.3
C7—C8—H8	108.1	C13—C17—H17	107.3
C14—C8—H8	108.1	C13—C18—H18A	109.5
C9—C8—H8	108.1	C13—C18—H18B	109.5
C11—C9—C8	111.2 (2)	H18A—C18—H18B	109.5
C11—C9—C10	115.2 (2)	C13—C18—H18C	109.5
C8—C9—C10	112.7 (2)	H18A—C18—H18C	109.5
C11—C9—H9	105.6	H18B—C18—H18C	109.5
C8—C9—H9	105.6	C10—C19—H19A	109.5
C10—C9—H9	105.6	C10—C19—H19B	109.5
C1—C10—C19	106.4 (2)	H19A—C19—H19B	109.5
C1—C10—C5	107.6 (2)	C10—C19—H19C	109.5
C19—C10—C5	112.5 (2)	H19A—C19—H19C	109.5
C1—C10—C9	111.6 (2)	H19B—C19—H19C	109.5
C19—C10—C9	110.8 (2)	C21—C20—C17	124.9 (3)
C5—C10—C9	107.9 (2)	C21—C20—H20	117.5
C9—C11—C12	112.4 (2)	C17—C20—H20	117.5
C9—C11—H11A	109.1	C20—C21—H21A	120.0
C12—C11—H11A	109.1	C20—C21—H21B	120.0
C9—C11—H11B	109.1	H21A—C21—H21B	120.0
C12—C11—H11B	109.1		
